# Supplementary material for: Limosilactobacillus reuteri DSM 17938 Produce Bioactive Components during Formulation in Sucrose
Source: Microorganisms. 2024 Oct 12;12(10):2058. doi: 10.3390/microorganisms12102058 (PMC11510291; doi:10.3390/microorganisms12102058)
Supplement: Supplementary file 1 [file microorganisms-12-02058-s001.zip › microorganisms-3216171-supplementary.pdf]

Supplementary Material

S. Table 1: Freeze drying protocol.

	**Loading**	**Freezing**	**Primary drying**			**Secondary drying**		
	Step 1	Step 2	Step 3	Step 4	Step 5	Step 6	Step 7	Step 8
**Time (hr: min)**		1	00:15	00:30	12:00	00:30	08:00	04:00
**Shelf temp. (°C)**	-40	-40	-35	-10	-10	-10	20	20
**Pressure (mbar)**			0.102	0.102	0.102	0.0100	0.0100	0.0100

S. Table 2: Dry weight of samples after freeze drying.

Sample	**Sucrose (g)**	**Glucose (g)**
**Directly frozen (DF)**	0.164	0.146
**Room temperature overnight (RT)**	0.133	0.140

S. Table 3: ^{1}H and ^{13}C NMR chemical shifts (δ, ppm) for the resonances from the α-glucan in the sucrose-RT and glucose-RT samples

	H1/C1	H2/C2	H3/C3	H4/C4	H5/C5	H67C6
→ 4)- α-D-Glc	5,34, 5.33, 5.30<br>102.5	3.59<br>74.2	4.05<br>75.9	3.65<br>80.3	3.98<br>72.5	3.72. 3.84*<br>63.6
→ 6)- α-D-Glc	(a)4.95, 4.94<br>(b)4.94, 4.95, 4,96	3.56<br>74.4	3.73<br>76.2	3.45<br>c	3.89<br>c	c

	100.9					

(a) ^{1}H chemical shifts of anomeric protons in the sucrose-RT sample
(b) ^{1}H chemical shifts of anomeric protons in the glucose-RT sample
(c) Not assigned unambiguously

Lyoconversion of freeze-drying protectant

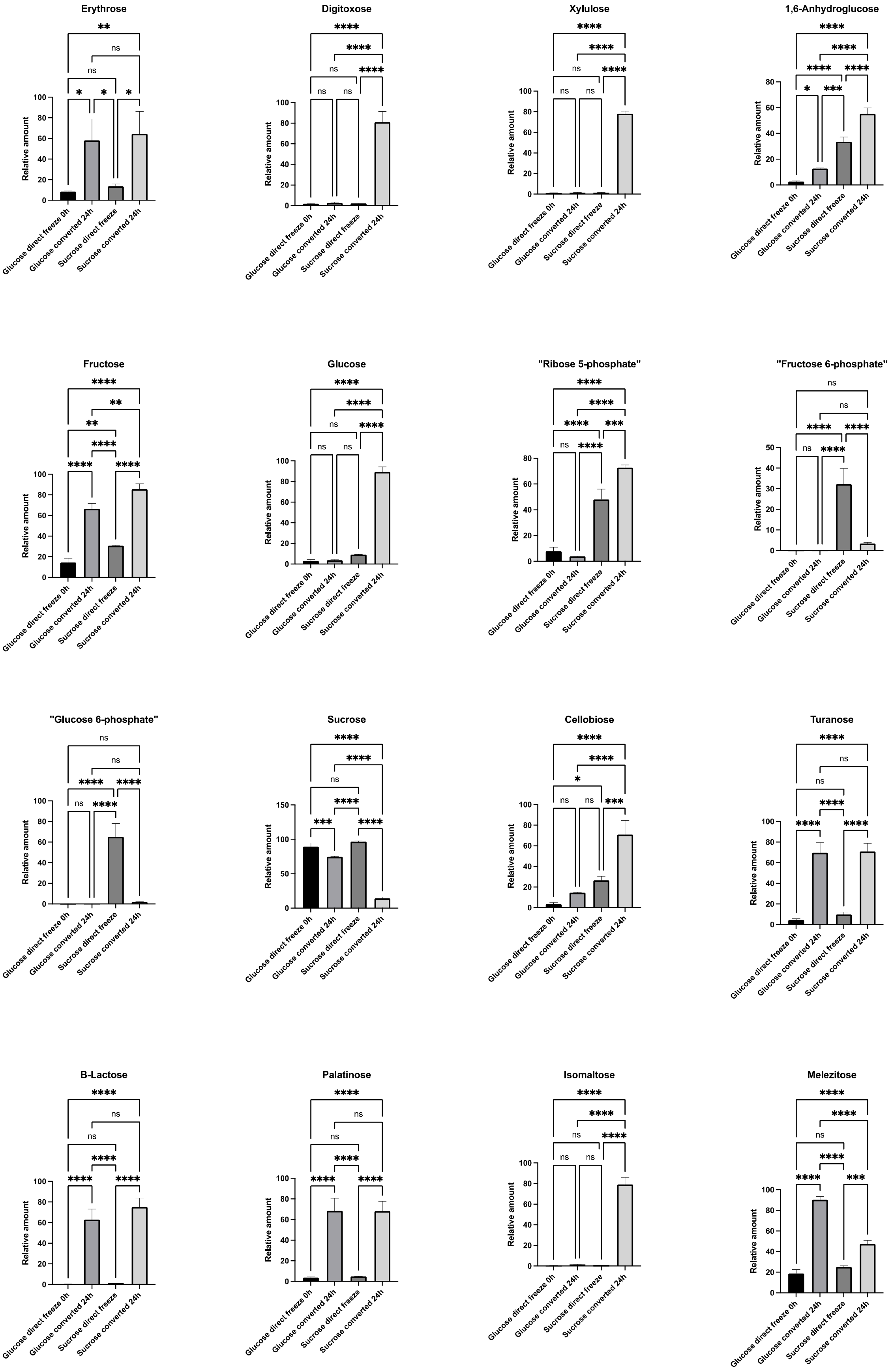

Lyoconversion of freeze-drying protectant

Supplementary Figure 1: Sugars detected by GCMS in the four types of samples. Values are relative, statistical analysis by One-Way ANOVA with Tukey's multiple comparisons test. N=3. Significance levels used were * $p < 0.05$; ** $p < 0.01$; *** $p < 0.001$, **** $p < 0.0001$, ns $P > 0.05$. Prism GraphPad version 9.0 was used for the statistical analyses.

Lyoconversion of freeze-drying protectant

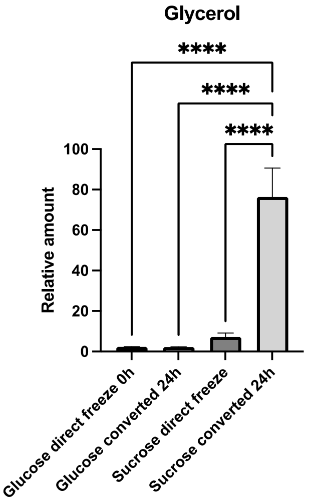

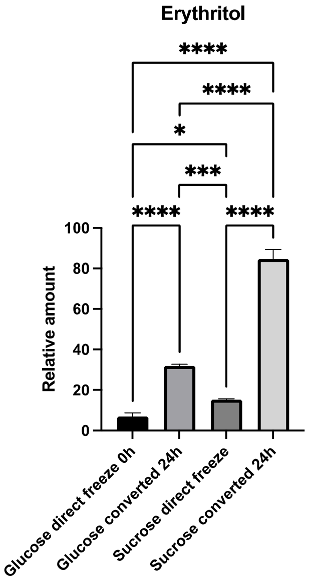

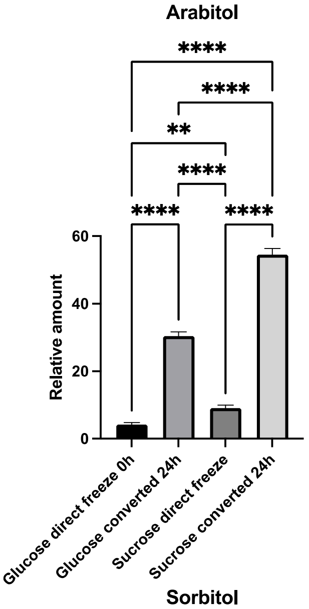

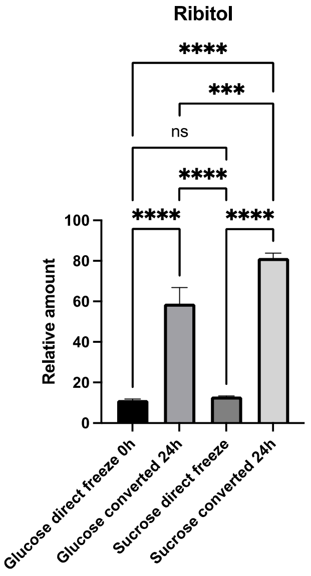

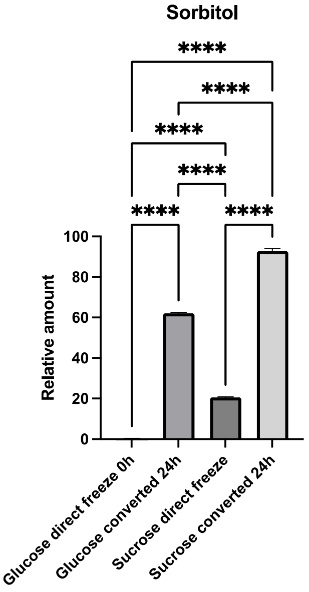

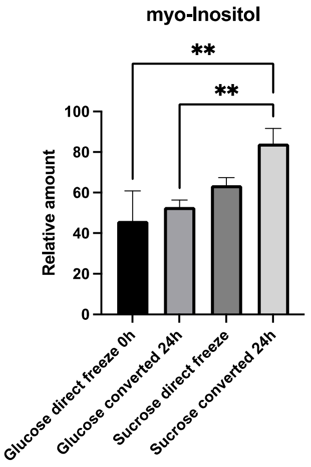

Lyoconversion of freeze-drying protectant

Supplementary Figure 2 Sugar alcohols detected by GCMS in the four different samples. Values are relative, statistical analysis by One-Way ANOVA with Tukey's multiple comparisons test. N=3. Significance levels used were * $p < 0.05$; ** $p < 0.01$; *** $p < 0.001$, **** $p < 0.0001$, ns $P > 0.05$. Prism GraphPad version 9.0 was used for the statistical analyses.

Lyoconversion of freeze-drying protectant

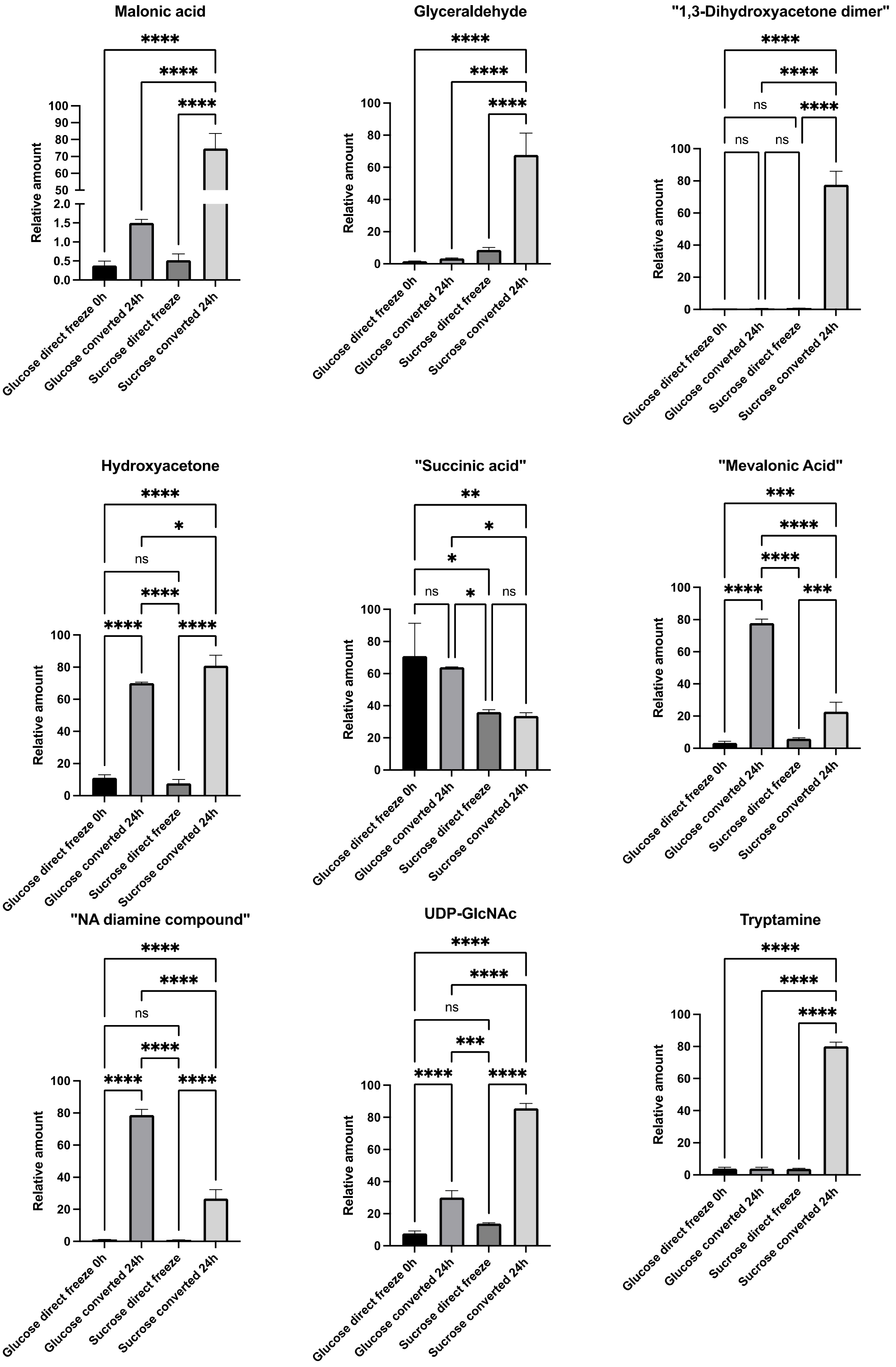

Lyoconversion of freeze-drying protectant

Supplementary Figure 3: Metabolites (not sugar, sugar alcohols or amino acids) detected by GCMS in the four different samples. Values are relative, statistical analysis by One-Way ANOVA with Tukey's multiple comparisons test. N=3. Significance levels used were * $p < 0.05$; ** $p < 0.01$; *** $p < 0.001$, **** $p < 0.0001$, ns $P > 0.05$. Prism GraphPad version 9.0 was used for the statistical analyses.

Lyoconversion of freeze-drying protectant

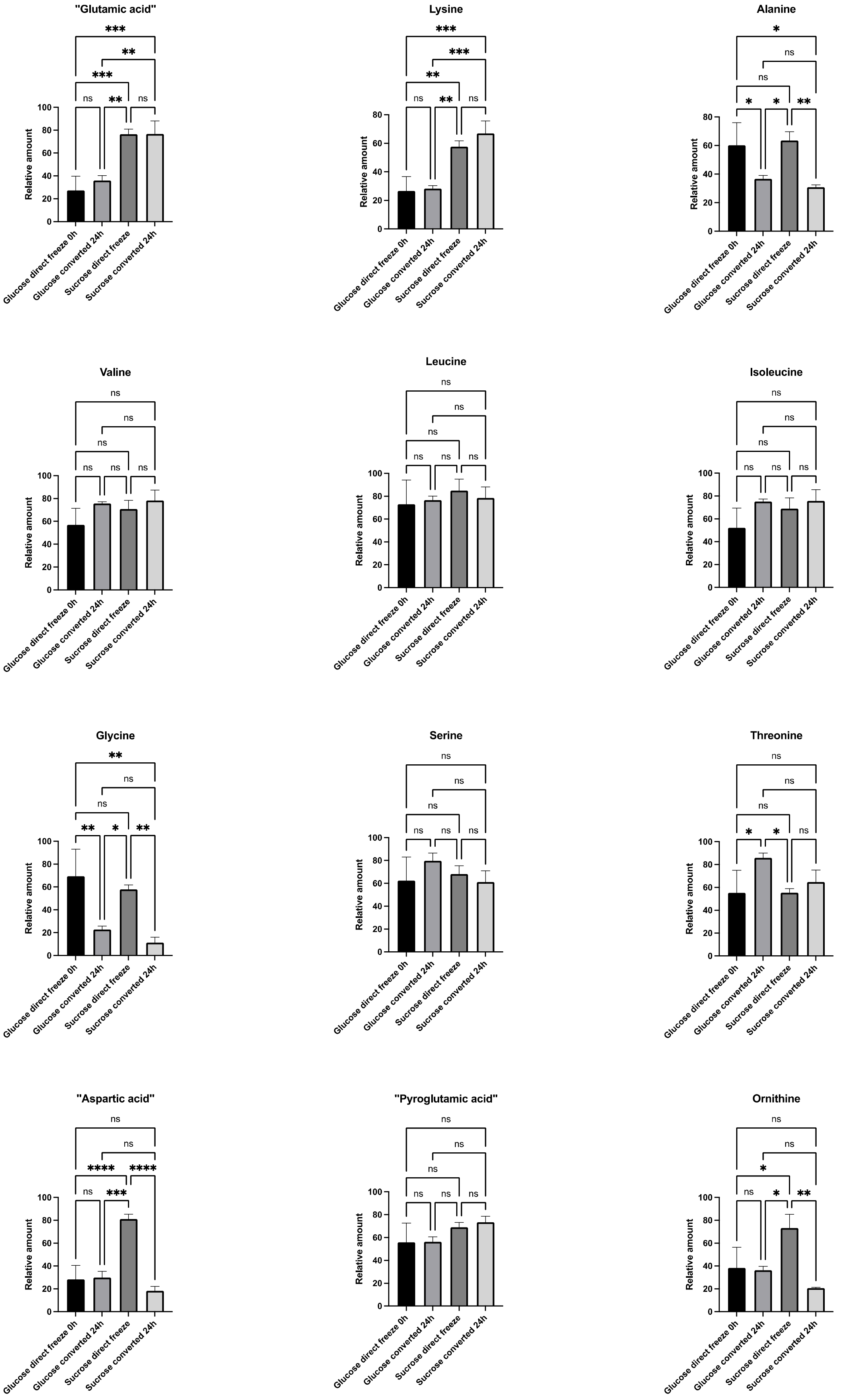

Lyoconversion of freeze-drying protectant

Supplementary Figure 4: Amino acids detected by GCMS in the four different samples. Values are relative, statistical analysis by One-Way ANOVA with Tukey's multiple comparisons test. N=3. Significance levels used were * $p < 0.05$; ** $p < 0.01$; *** $p < 0.001$, **** $p < 0.0001$, ns $P > 0.05$. Prism GraphPad version 9.0 was used for the statistical analyses.
